# Morphology of Macular Neovascularization in Age-Related Macular Degeneration Influences Treatment Requirement and Visual Outcome After 1 Year

**DOI:** 10.3390/jpm15060246

**Published:** 2025-06-11

**Authors:** Michael Grün, Kai Rothaus, Martin Ziegler, Albrecht Lommatzsch, Clemens Lange, Henrik Faatz

**Affiliations:** 1 Ophthalmology Department, St. Franziskus-Hospital, 48145 Münster, Germany; michael.gruen@augen-franziskus.de (M.G.);; 2 Achim-Wessing-Institute of Ophthalmologic Imaging, University of Essen, 45147 Essen, Germany; 3 Eye Center, University Hospital of Freiburg, 79106 Freiburg, Germany

**Keywords:** Optical Coherence Tomography Angiography (OCTA), age-related macular degeneration, retina

## Abstract

**Background/Objectives**: To evaluate the potential of Optical Coherence Tomography (OCT) and OCT angiography parameters in predicting treatment requirements and visual outcomes after one year in therapy-naïve eyes with neovascular age-related macular degeneration (nAMD). **Methods**: A retrospective study of 96 therapy-naïve eyes newly diagnosed with nAMD was carried out. All eyes received baseline OCT and OCTA. Follow-up OCT after initial upload was then carried out, involving three intravitreal injections (IVIs) with anti-Vascular Endothelial Growth Factor (anti-VEGF) at four-week intervals. OCT parameters, including intraretinal fluid (IRF), subretinal fluid (SRF), pigment epithelium detachment (PED), and central retinal thickness (CRT), were assessed qualitatively and quantitatively. Macular Neovascularization (MNV) morphology at baseline was described in terms of area, total vessel length, flow density, and fractal dimension. OCT and OCTA parameters were correlated with best corrected visual acuity (BCVA) and number of administered IVIs after 1 year of treatment. **Results**: Eyes with persistent subretinal fluid (SRF) or both intraretinal fluid (IRF) and SRF after upload showed a significantly higher need for IVIs (*p* < 0.01). Also, pigment epithelium detachment (PED) presence at baseline (*p* < 0.05), PED height at baseline (*p* < 0.01), PED presence after upload (*p* < 0.01), and PED height after upload (*p* < 0.01) were each correlated with a greater number of IVIs. Decrease in PED height during upload was accompanied by a lower number of IVIs (*p* < 0.01). All the aforementioned parameters had no influence on BCVA after 1 year (*p* > 0.05). Baseline central retinal thickness (CRT) was linked to worse BCVA at 12 months (*p* < 0.05), but not the number of IVIs. Follow-up CRT correlated with worse BCVA (*p* < 0.01) and more IVIs (*p* < 0.01), while CRT decrease was associated with better BCVA (*p* < 0.05) and fewer IVIs (*p* < 0.01) at 1 year. In OCTA, area and total vessel length of MNVs were correlated with BCVA after 1 year (*p* < 0.01) but had no influence on number of IVIs (*p* > 0.05). Flow density had no influence on either outcome parameter (*p* > 0.05). Fractal dimension was associated with BCVA (*p* < 0.01) and number of IVIs (*p* < 0.05) after 1 year. **Conclusions**: MNV area, vessel length, and fractal dimension in OCTA, along with fluid distribution in OCT at baseline and after follow-up, may serve as indicators of treatment needs and visual outcomes after one year. Further studies with longer observation periods and the use of deep learning models are needed to improve analyses and to verify the applicability of these findings to clinical practice.

## 1. Introduction

Age-related macular degeneration can be categorized into different disease stages. While early and intermediate stages may often be tolerated by patients, conversion into late-stage forms, such as geographic atrophy (GA) and neovascular AMD (nAMD), often marks the beginning of progressive vision loss. In developed countries, late-stage AMD is considered the main cause of blindness in adults. The risk of developing late-stage AMD grows with increasing age. In the European Union, due to demographic changes within an ageing population, the number of AMD patients is expected to increase by 15% by 2050 [1]. While treatment options for GA are still limited today, nAMD can be successfully treated with intravitreal injections (IVIs) of anti-VEGF (Vascular Endothelial Growth Factor) antibodies. However, treatment response may differ greatly between individuals. Prognostic assessments based on multimodal imaging are therefore crucial.

One of the most important imaging methods in identifying and describing MNV is Optical Coherence Tomography (OCT). This is a non-invasive technique that allows microscopically accurate depictions of retinal layers and pathological alterations. Guidelines suggest OCT scans for both diagnosis and treatment monitoring [2]. OCT is valuable both for the diagnosis and the monitoring of numerous retinal and systemic diseases, including macular holes [3], lamellar macular holes [4], AMD [5], disorders of the vitreoretinal interface [3], venous and arterial occlusions [6], diabetic retinopathy [7], inflammatory changes [8], inherited retinal diseases [9], glaucoma [10], drug-induced retinal toxicity [11], Alzheimer’s disease [12], multiple sclerosis [13], and others.

FA is the gold standard for visualizing retinal and choroidal vessels. However, this procedure has several drawbacks. It is an invasive technique that requires the intravenous administration of fluorescein. As a result, local side effects at the injection site and systemic complications can both occur, leading in very rare cases to an anaphylactic shock. During the imaging process, patients experience significant glare, and images must be captured immediately after fluorescein injection to obtain usable results. Leakage from MNV vessels causes blurring of the vascular structure, and changes in the retinal pigment epithelium (RPE) or hemorrhages can lead to signal attenuation or blockage. To avoid the aforementioned side effects, non-invasive methods are of particular interest. As well as imaging techniques, these also include other non-invasive tests; for example, measuring tear VEGF-levels as a biomarker for disease activity in nAMD [14,15].

Another relatively new non-invasive imaging modality is Optical Coherence Tomography Angiography (OCTA). This works by detecting movements of blood cells and transforming these movements into a grayscale image of vascular architecture in comparison to the surrounding tissue [16]. As well as its non-invasive nature, and in contrast to fluorescein angiography (FA), OCTA offers the advantage of a three-dimensional view that allows for a broader, more detailed picture about the morphology of MNV [17]. It is now even possible to visualize and monitor sub-clinical, non-exudative MNV that has not yet led to exudation and visual impairment [18,19]. Furthermore, the images may be translated into mathematical descriptions of MNV architecture, which was not possible when only OCT and FA imaging was used [20]. Told et al. found that various MNV morphology parameters in OCTA exhibit a dynamic pattern, as they show a response to initial therapy and a reactivation after the therapeutic effect fades. For instance, MNV size was found to typically shrink initially and then enlarge to above the baseline size just after the first anti-VEGF upload [21]. MNV morphology has therefore also gained interest for predicting the long-term course of the disease.

The purpose of this study was to identify OCT and OCTA parameters that have an influence on the course of the disease and could improve baseline assessment. There are different approaches to evaluate whether a treatment outcome is successful. From a patient-centered perspective, studies have shown that visual outcome remains the most important aspect to influence the patient’s quality of life [22,23]. From the perspectives of both the patient and the health care system, the frequency of administered IVIs also plays a crucial role, as every IVI involves great effort in terms of time, costs, and human resources. We therefore concentrated on best corrected visual acuity (BCVA) and number of injections as the main outcome parameters.

## 2. Materials and Methods

A retrospective, single-center study was performed at our clinic. The study was approved by Westphalia‒Lippe Medical Association and the University of Münster (approval code: 2021-066-f-S; approval date: 23 August 2021), and all patients gave written consent. Data processing and research was conducted in conformity with the views of the Declaration of Helsinki and its amendments.

### 2.1. Study Protocol

Eyes newly diagnosed with nAMD between November of 2016 and May of 2020 were consecutively included in this study. Eyes with any pathology other than nAMD were excluded, as well as eyes with coexistent pathologies like diabetic retinopathy or retinal vein occlusion. Also excluded from further analyses were eyes with deficient OCTA image quality, eyes in which we failed to detect MNV, and eyes which were not subject to a re-visit at 1 year. Diagnosis and classification of MNV type was made by ophthalmoscopy, FA, and SD-OCT. At initial visit, examination also included BCVA and OCTA. BCVA was assessed using decimal charts and then converted to logMAR for statistical analyses. A first follow-up visit after upload with three monthly IVIs of anti-VEGF was carried out to assess first treatment response. After 12 months, patients were re-visited to investigate the long-term course of the disease. Follow-up visits included a physical examination with BCVA, as well as ophthalmoscopy and SD-OCT imaging.

All diagnoses, clinical data, and findings were assessed retrospectively by 3 trained and certified graders at our reading center. If findings were unclear, cases were discussed by at least 2 of the graders. If findings were still uncertain, especially with regard to MNV depictions, these cases were dismissed and excluded from further analyses.

To analyze baseline characteristics and first treatment response after upload, a number of different parameters were assessed using OCT imaging. These included presence of retinal fluids (subretinal = SRF, intraretinal = IRF); pigment epithelium detachment (PED) presence and height; changes in IRF, SRF, and PED during upload; and change in central retinal thickness (CRT). Change in BCVA during upload was also assessed.

OCTA imaging at baseline was carried out with a swept-source OCTA PLEX^®^ Elite 9000 (Carl Zeiss Meditec, Dublin, CA, USA) machine. This device uses a wavelength of 1060 nm and has a scanning rate of 100.000 A-scans per second. It captures two consecutive B-scans of 500 A-scans within a 6 × 6 mm field of view. To enhance visualization and quantification of MNV, and create more accuracy and reliability, we enabled the automatic artifact suppression feature [24]. Segmentation from outer retina to choriocapillaris was used (ORCC: 0 µm from the outer plexiform layer to 49 µm below BM) [25,26]. When automatically drawn segmentations were incorrect, lines were properly adjusted manually. OCTA images were then exported to the program Fiji (National Institute of Mental Health, Bethesda, MD, USA), in which MNVs were framed and separated from the rest of the image. The vascular complex was then extracted using MatLab (Mathworks, Version R2014b; Natick, MA, USA). The MNV complex was skeletonized by multiscale calculation of the gradient field in the en-face image of the OCTA scan [27]. Four different parameters were selected to describe the MNV morphology: area, flow density, fractal dimension (FD), and total vessel length (sumL). Area represents the two-dimensional size of the MNV in the en-face view. Flow density represents the proportion of vascularization within a MNV. FD describes the complexity of a structure, and has been shown to be a significant parameter during anti-VEGF treatment [28]. SumL represents the total length acquired by summing all individual MNV vessels.

**Figure 1:**
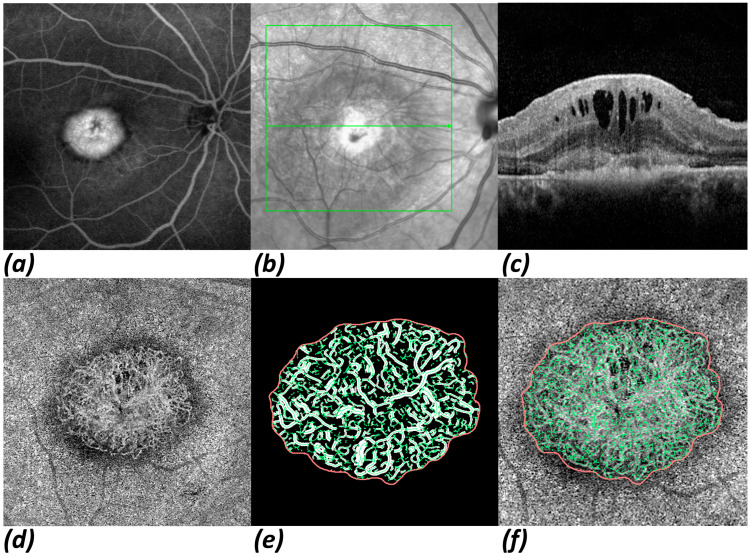
Multimodal imaging of a typical type 2 MNV, as presented in FA, NIR, OCT, and OCTA. Overviews of type 2 MNV in FA (**a**) and in near-infrared image (**b**), where an overlay of a green box, which represents the frame in which all SD-OCT slices were made, can be seen. The green line inside the box marks the position of the current OCT slice shown in (**c**). En-face view of the ORCC layer in OCTA (**d**). Enlarged views of binarized MNV (**e**) and skeletonized MNV (**f**). Note the epiretinal membrane as an additional finding. Although mild traction is apparent, intra- and subretinal fluid and retinal thickening is primarily caused by nAMD.

Figure 1 shows eye images obtained from a patient with an initial diagnosis of nAMD using multimodal imaging and processed OCTA data. The initial therapy response obtained by OCT imaging and the MNV morphology parameters acquired using the OCTA imaging and data extraction described above were correlated with BCVA after 1 year and the number of IVIs given within the first year of treatment.

### 2.2. Statistical Analysis

Statistical analyses were calculated with software R (Version 4.2.3, R Core Team 2023. R: A language and environment for statistical computing. R Foundation for Statistical Computing, Vienna, Austria). Threshold of significance was set to 5%. For this paper we used mean ± standard deviation to describe the distribution of a numeric variable and contingencies to describe categorial data. For correlation tests, we chose weighted linear regression models to identify the influence of independent variables (IVs) on dependent variables (DVs). IVs included all OCT and OCTA parameters, while BCVA and number of IVIs served as DVs. Weighted linear regression models were used to improve predictive accuracy by giving less weight to far outliers. Weights were introduced using the geometric mean weights of involved variables to reduce the influence of far outliers in the 10% and 90% percentiles (*p*_10_ and *p*_90_), so that:

$$w\left(x\right)=\exp-0.5\cdot {\left(\frac{\max\left\{0,\;t_{low}-x,\;x-t_{high}\right\}}{0.5\cdot \left(t_{high}-t_{low}\right)}\right)}^{2}\mathrm{where}$$

$$t_{low}=p_{10}-1.5\cdot \left(p_{90}-p_{10}\right)\;\mathrm{and}\;t_{high}=p_{90}+1.5\cdot \left(p_{90}-p_{10}\right)$$

The results of these regressions were given by the numbers of data (n), the regression estimates (beta), the 95% confidence (95% CI) intervals and the *p*-value of the univariable weighted linear regression.

## 3. Results

A total of 107 newly diagnosed nAMD eyes were recruited for the study. All received baseline examination. After applying exclusion criteria, 96 treatment-naïve eyes of 90 patients remained for further analyses. OCTA imaging was satisfactory in 80 eyes. The descriptive data shown in the following Table 1 refers to the total of 96 eyes examined. Of the total, 33.3% of eyes were of male patients and 66.7% of female patients, and mean age was 77.9 ± 7.1 years. Most of the neovascularizations were MNV type 1 (49.0%), followed by MNV type 2 (30.2%), and MNV type 3 (20.8%). BCVA was 0.58 ± 0.33 logMAR at baseline and 0.54 ± 0.40 logMAR at 1 year. An average of 8.26 ± 2.92 IVIs were administered throughout the first year of treatment.

At baseline, 8.3% of eyes showed only IRF and 41.7% of eyes showed only SRF, while 50.0% had both IRF and SRF. A total of 65.5% of eyes had PED, with an average height of 177 ± 202 μm. Average central retinal thickness was 469 ± 177 μm. A total of 80 eyes with adequate OCTA quality were eligible for further analysis of OCTA parameters at baseline. On average, MNV area size was 1.56 ± 1.84 mm² while average flow density was 41.2 ± 5.2%. Average fractal dimension was 1.292 ± 0.142 and total vessel length was 18.2 ± 21.7 mm.

After upload, IRF disappeared in 40.6% of eyes but persisted in 17.7% of eyes; in addition, 2.1% of eyes had new IRF and 39.6% remained stable without IRF. In 64.6% of eyes SRF disappeared, and in 27.1% of eyes SRF persisted. PED completely disappeared in 22.3% of eyes and persisted in 42.6%, while 6.4% of eyes developed new PED. On average, a decrease in PED of 47.0 ± 48.0 μm and a decrease in CRT of 83.3 ± 26.5 μm was seen.

All OCT and OCTA baseline parameters and first treatment responses obtained in OCT, as well as their respective influences on BCVA and number of IVIs after 1 year, are displayed in Table 2 and Table 3.

Eyes with persistent SRF or with both IRF and SRF after upload showed a significantly higher need for IVIs within the first year of treatment (*p* < 0.01). Furthermore, PED parameters were correlated with greater numbers of IVIs; these parameters included PED presence at baseline (*p* < 0.05), PED height at baseline (*p* < 0.01), PED presence after upload (*p* < 0.01), and PED height after upload (*p* < 0.01). Accordingly, a greater decrease in PED height during upload was associated with fewer IVIs (*p* < 0.01). However, these parameters had no influence on BCVA after 1 year (*p* > 0.05). CRT at baseline was associated with a worse BCVA (*p* < 0.05) but had no influence on number of IVIs (*p* < 0.05). CRT at follow-up was correlated with both a worse BCVA (*p* < 0.01) and a higher need for IVIs (*p* < 0.01). A greater decrease in CRT during upload also had an impact on both a better BCVA (*p* < 0.05) and fewer IVIs (*p* < 0.01).

Regarding OCTA morphology parameters at baseline, both area size and the total vessel length of MNV lesions were significantly correlated with BCVA after 1 year (*p* < 0.01) but had no influence on the number of IVIs (*p* > 0.05). Flow density had no influence on either outcome parameter (*p* > 0.05). Fractal dimension was associated with both BCVA (*p* < 0.01) and number of IVIs (*p* < 0.05) after 1 year.

## 4. Discussion

Disease-activity and prognostic biomarkers rely primarily on OCT findings. These include distribution of retinal fluid at baseline, such as intraretinal fluid being a prognostic factor for a worse visual outcome [29], as well as persistent retinal fluid after initial therapy [30]. Applying a machine learning approach to OCT scans, Bogunovic et al. found subretinal fluid to be the most important predictor of IVI requirement within the first two years of therapy [31]. Another important biomarker is CRT, as it is especially suitable for disease control in clinical studies because quantification is relatively easy. Accordingly, reduction in CRT is associated with better visual outcome [32]. PED at baseline also seems to play a crucial role in predicting persistent disease activity and visual decline [29,30]. Choroidal assessment has also gained interest, as parameters such as choroidal thickness (CT) and choroidal vascularity index (CVI) have been shown to be linked with disease progression [33,34,35]. In addition, several other OCT findings have been shown to be associated with disease outcome; these include outer retinal tubulations [36], integrity of ellipsoid zone [37], hyperreflective foci [38], and subretinal drusenoid deposits [39].

This study confirms some of these findings. In our cohort, eyes with subretinal fluid at baseline and eyes with persistent fluid after upload both showed a significantly higher need for IVIs within the first year. Also, the presence of PED and its response to therapy were associated with higher therapy requirements. CRT at baseline and at upload, as well as its response to initial therapy, were particularly important for visual outcome after 1 year.

The IRF, SRF, and PED visible in OCT are secondary consequences of the disease, caused by the invasively growing MNV and the instability of its vascular walls. By directly examining the primary cause—the MNV itself—in OCTA, we hope to identify additional and better biomarkers that provide insights into disease progression. OCTA facilitates a more detailed depiction of MNV due to its three-dimensional view [40]. With OCTA imaging, the morphology of MNV can be mathematically described more precisely, potentially enabling identification of new parameters that can predict disease outcome [20]. MNV morphology has therefore gained interest in also predicting the long-term course of the disease.

Previous studies have described an association between the MNV architecture in OCTA and outcome parameters such as IVI requirement and BCVA. We recently demonstrated that area size, total vessel length, and flow value are all associated with reduced BCVA after 1 year, and that flow value and vessel caliber both impact IVI requirement during the first year of treatment [41]. Faghihi et al. reported that mean vessels percentage area and fractal dimension were predictors of a better visual outcome after 3 months and 12 months [42].

However, these studies used RTVue XR Avanti with AngioVue™ (Optovue, Inc., Freemont, CA, USA) for OCTA scans [41,42]. Just as with OCT, there are different devices available to carry out OCTA scans, based on different imaging and data-processing techniques. In a previous study, it was shown that RTVue XR Avanti scans were comparable with PLEX^®^ Elite 9000 scans in terms of MNV size parameters. However, flow density and fractal dimension showed distinct differences between the two devices [43]. In this study, our further aim was therefore to investigate MNV morphology characteristics in a cohort using PLEX^®^ Elite 9000 as an OCTA device.

Area and total vessel length are two morphological parameters that describe the size of MNV. In this cohort, both parameters were associated with worse visual outcome after 1 year but did not influence the number of IVIs needed within the first year. Both these findings are in accordance with previously reported results [41]. Some MNV morphology characteristics like vascular density and fractal dimension have shown to be promising parameters to assess MNV activity [44]. FD describes the complexity of vessels. In our cohort, FD seemed to be an important parameter for predicting the course of the disease within the first year. A higher FD was associated with a poorer visual outcome and more IVIs needed. Interestingly, this is in contrast with Faghihi et al., who proposed that FD was a predictor for a better visual outcome [42].

Flow density refers to the proportion of pixels with a flow signal in relation to the total amount of pixels in the displayed MNV area. In this cohort, there was no correlation between flow density and visual outcome or therapy burden after 1 year. However, this finding may be biased because only erythrocytes moving within a detectable pace can be captured and eventually interpreted as vessels, so “no flow” does not imply there are actually no vessels [45]. Furthermore, if erythrocytes are moving towards or away from the detector, flow may not be captured. In conclusion, flow density as a parameter has several limitations, which is why it is crucial to include other parameters to describe MNV morphology.

Although we assessed some of the most important OCT parameters addressing outcome prediction, other parameters with potential prognostic value, such as choroidal thickness or hyperreflective foci, were not assessed in this study. As with the OCT parameters, it would have been desirable to also detect a treatment response in the OCTA parameters, reflecting changes in MNV vascular morphology, and to correlate these with the clinical course. This was not possible in this retrospective analysis but it remains a goal for future studies, so that better biomarkers may be identified based on the direct treatment response of MNV vessels. Further limitations of this study include its retrospective character and single-center design. A central limitation arises from the resolution capacity of OCT-A imaging, which is especially important when analyzing fine vascular structures, such as capillaries. Furthermore, the portrayed parameters of the MNV in OCTA, namely, area, total vessel length, FD, and flow density, only represent the perfused portion of the MNV in a given two-dimensional segmentation. Moreover, in the present study, the MNV had to be manually outlined, a process which always involves some subjectivity. The process of automated vessel skeletonization is thus limited because vessels may be incorrectly identified. Hence, in future studies, the use of deep learning models to correlate as many retinal and MNV parameters as possible could improve analysis of predictive factors for visual outcomes and treatment requirements. These more-comprehensive analyses could also include further assessments exceeding imaging techniques. For instance, molecular biomarkers like tear VEGF-levels could be beneficial in assessing disease activity and, potentially, improving prediction of long-term outcomes [14,15].

## 5. Conclusions

In conclusion, we investigated OCT parameters at baseline and after initial upload, as well as baseline MNV characteristics in OCTA, to predict therapy requirement and visual outcome after 1 year. In OCT, SRF, and PED, presence and height, as well as response to initial therapy, were the most important parameters in respect of the number of IVIs needed. CRT was especially important in predicting visual outcome after 1 year. In OCTA, we found that morphology parameters describing lesion size were associated with worse visual outcome, in accordance with previous studies. FD as a parameter describing MNV complexity was associated with both worse visual outcome and higher therapy burden, the latter being in contrast with previous findings. We saw no influence of flow density on outcome after 1 year. Not only the morphological findings of the retina in OCT at initial diagnosis, but also the initial treatment response to anti-VEGF therapy, including changes in existing pathologies, provide insights into the further clinical course of nAMD. OCTA-based vascular parameters also exhibit characteristic prognostic features that enhance the prediction of disease progression. Further studies with longer observation periods and the use of deep learning models are needed to improve analyses and to verify the applicability of these findings to clinical practice.

## Figures and Tables

**Table 1:** General clinical data.

Age	77.9 ± 7.1 Years (*n* = 96)
Gender	Male 33.3% (*n* = 32), Female 66.7% (*n* = 64)
Laterality	Right eye 55.2% (*n* = 53), left eye 44.8% (*n* = 43)
MNV type	Type 1 49.0% (*n* = 47), type 2 30.2% (*n* = 29), type 3 20.8% (*n* = 20)
BCVA (logMAR) Baseline At 1 year	0.58 ± 0.33 (*n* = 96) 0.54 ± 0.40 (*n* = 96)
Number of IVIs at 1 year	8.26 ± 2.92 (*n* = 96)

General clinical data of the full study cohort of 96 eyes.

**Table 2:** All assessed OCT and OCTA parameters and their influence on BCVA after 1 year.

	Unit	n	Beta	95% CI	*p* Value
OCT parameters					
CRT					
baseline	200 µm	96	0.110	[0.021; 0.198]	<0.05
after upload	200 µm	94	0.165	[0.043; 0.287]	<0.01
decrease during upload	10%	94	−0.035	[−0.064; −0.005]	<0.05
PED					
presence at baseline	present	96	0.116	[−0.052; 0.283]	0.179
height at baseline	200 µm	96	0.072	[−0.007; 0.150]	0.078
presence after upload	present	94	0.001	[−0.159; 0.161]	0.988
height after upload	200 µm	94	0.079	[−0.027; 0.185]	0.148
height decrease during upload	10%	61	−0.001	[−0.021; 0.020]	0.937
IRF after upload	present	93	0.125	[−0.132; 0.383]	0.343
SRF after upload	present	93	0.111	[−0.101; 0.322]	0.309
IRF and SRF after upload	present	93	0.104	[−0.192; 0.399]	0.493
OCTA parameters					
MNV area	1 mm	80	0.096	[0.050; 0.142]	<0.01
MNV total vessel length	20 mm	80	0.153	[0.074; 0.232]	<0.01
MNV flow density	10%	80	−0.098	[−0.275; 0.078]	0.279
MNV fractal dimension		80	1.017	[0.403; 1.631]	<0.01

All assessed OCT and OCTA parameters and their correlation with BCVA after 1 year are displayed. The table shows the units of each parameter (unit), the numbers of data (n), the regression estimates (beta), the 95% confidence (95% CI) intervals, and the *p*-values of the univariable weighted linear regression.

**Table 3:** All assessed OCT and OCTA parameters and their influence on number of IVIs at 1 year.

	Unit	n	Beta	95% CI	*p* Value
OCT parameters					
CRT					
baseline	200 µm	96	0.493	[−0.166; 1.152]	0.145
after upload	200 µm	94	1.741	[0.864; 2.619]	<0.01
decrease during upload	10%	94	−0.346	[−0.562; −0.130]	<0.01
PED					
presence at baseline	present	96	1.413	[0.211; 2.614]	<0.05
height at baseline	200 µm	96	0.833	[0.275; 1.391]	<0.01
presence after upload	present	94	2.502	[1.419; 3.584]	<0.01
height after upload	200 µm	94	1.851	[1.143; 2.559]	<0.01
height decrease during upload	10%	61	−0.295	[−0.435; −0.154]	<0.01
IRF after upload	present	93	3.028	[1.614; 4.441]	0.175
SRF after upload	present	93	1.205	[−0.516; 2.925]	<0.01
IRF and SRF after upload	present	93	3.375	[1.403; 5.347]	<0.01
OCTA parameters					
MNV area	1 mm	80	0.193	[−0.162; 0.549]	0.290
MNV total vessel length	20 mm	80	0.397	[−0.204; 0.998]	0.199
MNV flow density	10%	80	−0.819	[−2.067; 0.430]	0.202
MNV fractal dimension		80	5.037	[0.540; 9.534]	<0.05

All assessed OCT and OCTA parameters and their correlation with number of IVIs at 1 year are displayed. The table shows the units of each parameter (unit), the numbers of data (n), the regression estimates (beta), the 95% confidence (95% CI) intervals, and the *p*-values of the univariable weighted linear regression.

## Data Availability

The datasets presented in this article are not readily available. Requests to access the datasets should be directed to M.G.

## Abbreviations

The following abbreviations are used in this manuscript:

OCT	Optical coherence tomography
OCTA	Optical coherence tomography angiography
nAMD	Neovascular age-related macular degeneration
IVI	Intravitreal injection
VEGF	Vascular endothelial growth factor
IRF	Intraretinal fluid
SRF	Subretinal fluid
PED	Pigment epithelium detachment
MNV	Macular neovascularization
BCVA	Best corrected visual acuity
CRT	Central retinal thickness
FA	Fluorescence angiography
FD	Fractal dimension
sumL	Total vessel length
IV	Independent variable
DV	Dependent variable
CT	Choroidal thickness
CVI	Choroidal vascularity index
